# Supplementary material for: Content-rich biological network constructed by mining PubMed abstracts
Source: BMC Bioinformatics. 2004 Oct 8;5:147. doi: 10.1186/1471-2105-5-147 (PMC528731; doi:10.1186/1471-2105-5-147)
Supplement: Additional File 2 — The original results of the above study (non-essential files are deleted to keep the file size under the limit set by BMC bioinformatics). [file 1471-2105-5-147-S2.bz2 › chilibotAdditionalFile2/dip10/135ID7784054E480/html/TP53_RAF1.html]

 


 **TP53** and **RAF1** 
  
Found 9 abstracts in PubMed, retrieved 9.  
 

 What does Google say? 
 PDF only 
| .edu only 

---

**Interactive relationship** (e.g. stimulation, inhibition, etc)

**Inhibitory relationship**- Inhibition of Hsp90, a chaperone protein a protein that helps other proteins avoid misfolding pathways that produce inactive or aggregated states, leads to depletion of important oncogenic proteins, including Raf 1  [ **RAF1** ]  and mutant p53 also known as  **TP53** .  Ref: 10564678 J Natl Cancer Inst, 1999
**Neutral relationship**

**Non-interactive relationship** (e.g. studied together, co-existance, homology, etc.)

- No association between radiosensitivity and  **TP53**  status, G1 arrest or protein levels of p53, myc, ras or raf  [ **RAF1** ]  in human melanoma lines.  Ref: 10528923 Int J Radiat Biol, 1999
- However, in the ovarian cancer cell lines that have a mutant  **TP53** , the cytotoxicity induced by 60 nM paclitaxel exhibited the same relationship to Raf 1  [ **RAF1** ]  kinase activity as previously observed in cervical tumor cell lines.  Ref: 10854551 Oncol Rep< MedlineTA>Oncol Rep, 1999
- In the two cell lines that possess a wild type  **TP53** , the relationship between Raf 1  [ **RAF1** ]  kinase activity and paclitaxel resistance was different from that observed in the cervical tumor cell lines.  Ref: 10854551 Oncol Rep< MedlineTA>Oncol Rep, 1999
- Sustained activation of Ras Raf  [ **RAF1** ]  mitogen activated protein kinase cascade by the tumor suppressor p53  [ **TP53** ] .  Ref: 10890907 Proc Natl Acad Sci U S A, 2000
- CONCLUSIONS Differences in radiosensitivity among melanoma lines cannot be elucidated by  **TP53**  status, differences in G1 arrest or different levels of p53, myc, ras or raf  [ **RAF1** ]  proteins.  Ref: 10528923 Int J Radiat Biol, 1999
- Tumor suppressor p53  [ **TP53** ]  induction in response to cellular stresses activates the mitogen activated protein kinase MAPK cascade through pathways involving Ras and RAF  [ **RAF1** ]  p53 s ability to activate this pathway is dependent on p53 mediated transcription.  Ref: 11296226 EMBO J, 2001
- PURPOSE First, to investigate whether  **TP53**  status and or radiation induced G1 arrest are associated with radiosensitivity, and, second, to detect possible associations between protein levels of p53, myc, ras or raf  [ **RAF1** ]  and radiosensitivity and to investigate whether hypoxia induced changes in the levels of these proteins are related to hypoxia induced changes in radiosensitivity in human melanoma lines.  Ref: 10528923 Int J Radiat Biol, 1999
